# Ileostomy for Refractory Immune Checkpoint Inhibitor-Associated Colitis: A Case Report and Literature Review

**DOI:** 10.7759/cureus.93442

**Published:** 2025-09-28

**Authors:** Koji Daito, Masahiro Haeno, Issei Umeda, Kazuki Ueda, Junnichiro Kawamura

**Affiliations:** 1 Department of Surgery, Kindai University Faculty of Medicine, Osaka, JPN

**Keywords:** case report, checkpoint inhibitor colitis, diverting loop ileostomy, ibd, ileostomy, immune checkpoint inhibitors, immune-related adverse event, irae colitis, refractory colitis, ulcerative colitis

## Abstract

Immune checkpoint inhibitors (ICIs) are widely used to treat malignant tumors and have transformed cancer treatment; however, immune-related adverse events (irAEs) can occur due to excessive immune responses. Among these, immune checkpoint inhibitor-related colitis (irAE colitis) presents with symptoms resembling ulcerative colitis, for which high-dose corticosteroids and anti-TNF-alpha antibodies are recommended. Surgical treatment has not been well established for cases refractory to medical therapy. We report a case of a 64-year-old male patient with PD-L1-positive recurrent lung adenocarcinoma who developed hematochezia and diarrhea eight weeks after initiating pembrolizumab, leading to a diagnosis of irAE colitis. Despite treatment with corticosteroids, infliximab, mesalazine, and leukocytapheresis, the colitis remained refractory. Due to the patient's poor performance status, proctocolectomy and subtotal colectomy were not indicated, and a diverting loop ileostomy was performed to rest the colon. Following surgery, abdominal symptoms resolved, allowing chemotherapy to resume three months later. Stoma closure was not possible due to persistent mucosal lesions on colonoscopy, but no symptoms of colitis were observed, and nivolumab was administered from the 18^th^ month post surgery. However, the treatment was ineffective, and the patient succumbed to the primary disease two years after surgery. This case highlights the potential role of diverting loop ileostomy in managing refractory irAE colitis, though further discussion is needed regarding surgical strategies and the feasibility of resuming ICIs following colitis development.

## Introduction

Immune checkpoint inhibitors (ICIs) are widely used to treat malignant tumors and have transformed cancer treatment. By enhancing T-cell activation, ICIs effectively target cancer. However, excessive immune activation can lead to T-cell infiltration into various organs, triggering immune-related adverse events (irAEs) [1]. Immune checkpoint inhibitor-related colitis (irAE colitis) presents symptoms similar to ulcerative colitis. Guidelines recommend high-dose corticosteroids for grade 2 or higher irAE colitis and additional anti-TNF-α antibodies for steroid-refractory cases [2,3]. However, when medical therapy is ineffective, the role of surgical treatment remains unclear. We present a case of irAE colitis that was refractory to conservative treatment and clinically improved with a diverting loop ileostomy.

This article was previously posted as a preprint on the Research Square server on May 24, 2022 (DOI for the preprint version: https://doi.org/10.21203/rs.3.rs-1664360/v).

## Case presentation

**Figure 1:**
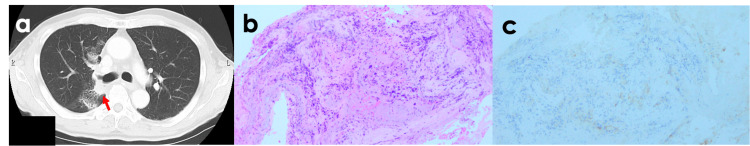
Radiological and histopathological findings at recurrence (a) Axial CT image obtained 18 months after initial treatment, showing a recurrent lung lesion (arrow). (b) Hematoxylin and eosin (HE) staining of the biopsy specimen, demonstrating glandular structures characteristic of adenocarcinoma. (c) Immunohistochemical staining for PD-L1, showing membranous positivity in tumor cells. The Tumor Proportion Score (TPS) was 25%–49%. The arrow in (a) indicates the site of the recurrent lesion. HE and PD-L1 staining confirmed the diagnosis of recurrent lung adenocarcinoma. PD-L1 immunohistochemistry revealed TPS of 25%–49%, indicating PD-L1 positivity and eligibility for anti-PD-1 therapy.

**Figure 2:**
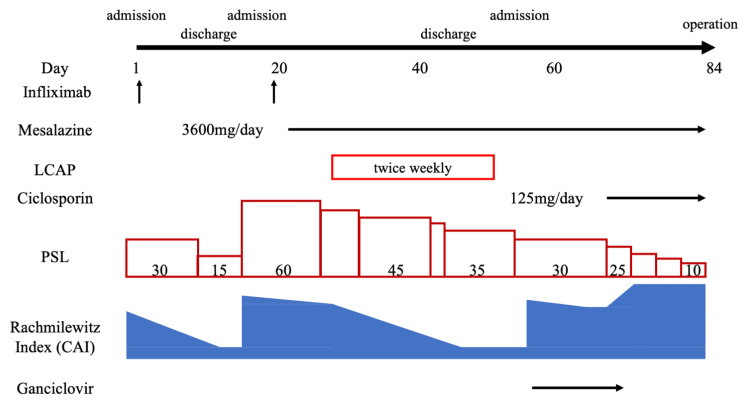
Course of the disease from admission to surgery LCAP: leukocytapheresis; PSL: prednisolone

This case involved a 64-year-old male patient who was initially diagnosed with stage IIIA lung adenocarcinoma (cT1bN2M0) and underwent chemoradiotherapy (CRT) at a different hospital. The detailed tumor location and CRT regimen were not documented in the referral records. Surgical resection was not performed, likely due to mediastinal lymph node involvement. Eighteen months later, a new pulmonary lesion was detected in a different lobe, which was pathologically confirmed as adenocarcinoma and diagnosed as recurrent lung adenocarcinoma. Immunohistochemical analysis of PD-L1 showed a tumor proportion score (TPS) of 25%-49%. Based on the PD-L1 status, ICI therapy with pembrolizumab was initiated (Figure 1). In the eighth week after treatment initiation, hematochezia developed, followed by diarrhea in the ninth week. Despite receiving prednisolone for a diagnosis of irAE colitis, the patient's symptoms did not improve, necessitating transfer to our hospital for further treatment. The clinical course following the onset of colitis is summarized in Figure 2. A clinical evaluation was performed using the Rachmilewitz index (Clinical Activity Index, CAI), in accordance with inflammatory bowel disease assessment criteria [4].

**Figure 3:**
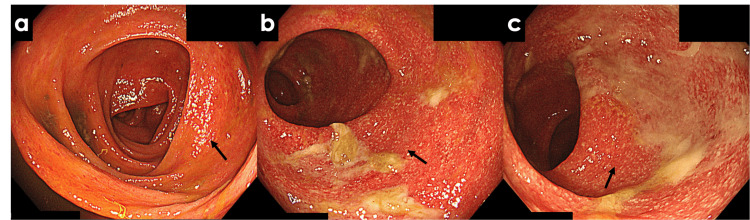
Colonoscopy images before treatment initiation (a) Transverse colon: Diffuse mucosal erythema (highlighted by arrow); (b) Sigmoid colon: Sporadic erosions and mucopurulent adherence (arrows); (c) Rectum: Mucosal erythema and mucopurulent adherence (arrows). Arrows indicate the areas of interest, including mucosal inflammation and purulent exudates.

The CAI score at admission was eight. Colonoscopy revealed diffuse mucosal erythema and erosions extending from the rectum to the transverse colon, along with mucopurulent adherence in the rectum and sigmoid colon (Figure 3). The findings resembled ulcerative colitis. Histopathological examination confirmed cryptitis, crypt abscesses, and architectural distortion, consistent with ulcerative colitis. Based on these findings, a diagnosis of irAE colitis was established, and treatment with infliximab and prednisolone (30 mg/day) commenced. Although the patient initially responded well, with symptoms improving and discharge occurring on day 10 (CAI score: 2), he experienced recurrent abdominal pain and diarrhea, necessitating readmission (CAI score: 7). Additional treatment with infliximab, mesalazine (3600 mg/day), and increased prednisolone (60 mg/day) provided no significant symptomatic relief. Leukocytapheresis (twice weekly) was introduced, yielding temporary improvement, but symptoms recurred after one month (CAI score: 10). Ciclosporin was added to the regimen, and ganciclovir was initiated due to cytomegalovirus positivity.

**Figure 4:**
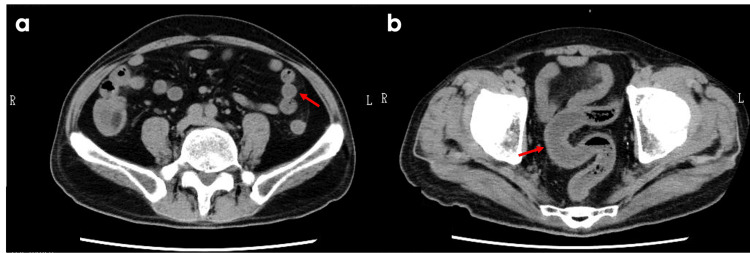
Computed tomography (CT) images after medical treatment (a) Axial CT image showing bowel wall edema localized in the small intestine (highlighted by red arrows), with no evidence of gastrointestinal perforation. (b) Axial CT image demonstrating continuous edema extending to the colon and rectum (arrows). Arrows indicate areas of bowel wall thickening and edema in both the small and large intestines.

**Figure 5:**
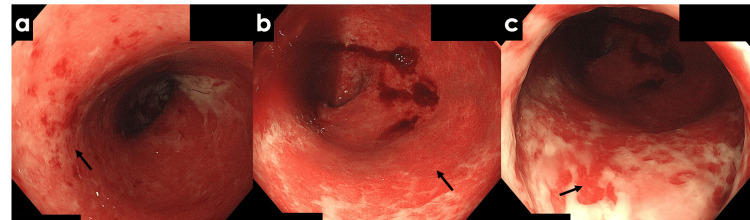
Colonoscopy images after medical treatment (a) Sigmoid colon: Persistent mucosal redness and mucopurulent adherence (highlighted by arrows); (b) Rectosigmoid colon: Unchanged mucosal erosions and exudate (arrow); (c) Lower rectum: Mucosal erythema and erosion with mucopurulent covering (arrow). Arrows show sites with no significant endoscopic improvement post treatment.

Computed tomography (CT) imaging showed no gastrointestinal perforation (Figure 4), yet colonoscopy revealed persistent mucosal lesions and ongoing abdominal symptoms (Figure 5). We considered the indications for proctocolectomy or subtotal colectomy based on treatment strategies for refractory ulcerative colitis; however, given the patient's poor performance status (PS: 3), these procedures were deemed too invasive. Instead, a laparoscopic diverting loop ileostomy was performed to allow colonic rest.

**Figure 6:**
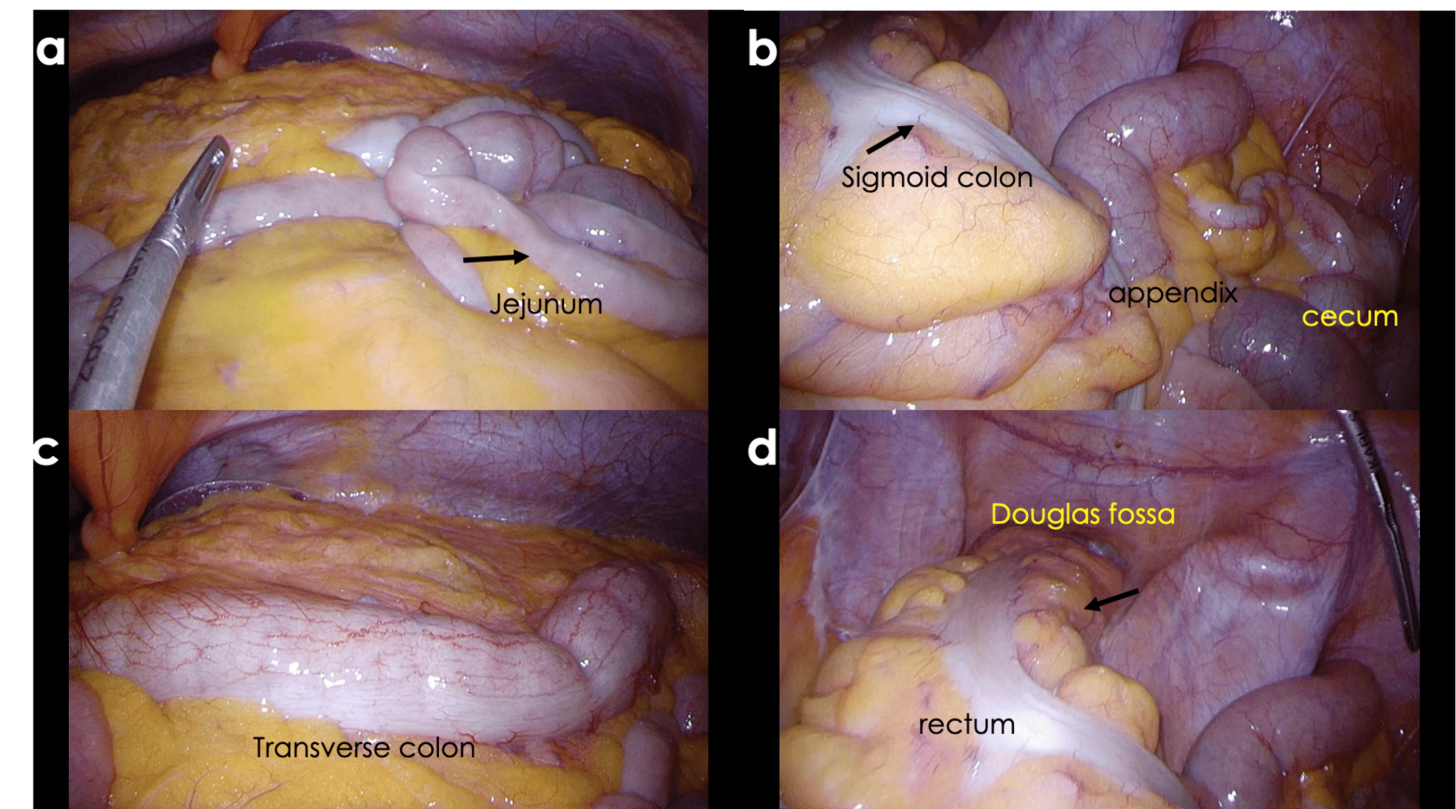
Intra-abdominal findings during surgery (a) The jejunum appears slightly edematous (arrow). (b) The sigmoid colon, cecum, and appendix without perforation or ascites; mild edema in the sigmoid colon (arrows). (c) The transverse colon appears nearly normal (no arrows). (d) Edema in the mesorectum is evident (arrow); no ascites in the Douglas fossa. Arrows indicate locations of edema and inflammation, supporting surgical observations.

The surgery was performed laparoscopically. A camera port was placed at the umbilicus, the abdominal cavity was searched, and no ascites or abscess was found. The gastrointestinal tract showed edema throughout; however, no abnormalities were found on the serosal surface (Figure 6). A diverting loop ileostomy was performed using the ileum, 30 cm from the terminal ileum.

**Figure 7:**
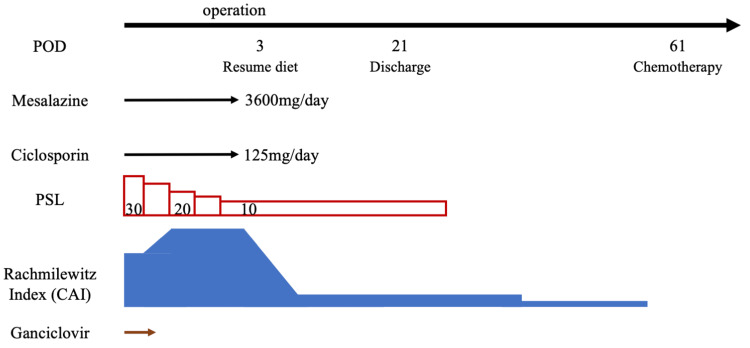
Course of the disease in the perioperative period POD: postoperative day; PSL: prednisolone; CAI: Clinical Activity Index

The perioperative course is shown in Figure 7. Postoperatively, abdominal symptoms improved, and the patient resumed oral intake by postoperative day (POD) 3. Over time, general condition, abdominal pain, and hematochezia improved (CAI score: 3). Prednisolone therapy continued, and the patient was discharged 21 days after surgery.

Chemotherapy with pemetrexed was initiated on POD 61. Prednisolone was tapered to 10 mg/day by POD 22 and was discontinued shortly after. Chemotherapy with pemetrexed was initiated approximately two months after surgery, followed by docetaxel and TS-1. Prednisolone was tapered to 10 mg/day by POD 22 and discontinued shortly thereafter. Stoma closure could not be performed because the mucosal lesions did not improve on colonoscopy; nevertheless, the patient remained free of irAE colitis symptoms. Nivolumab was reintroduced 18 months after surgery. The patient developed grade 1 colitis shortly after reinitiation, which resolved with conservative management, allowing treatment to continue. Nivolumab was continued for approximately four months, but radiological assessment revealed disease progression, including worsening of spinal metastasis. Based on Response Evaluation Criteria in Solid Tumors (RECIST) criteria [5], the treatment was deemed ineffective and discontinued. No further anticancer therapies were administered, and the patient received the best supportive care until death from the primary disease two years after surgery.

## Discussion

The first ICIs for metastatic melanoma were approved by the U.S. Food and Drug Administration (FDA) in 2011, and since then, ICIs have become the standard medical treatment for various cancers. However, several irAEs have been reported [1]. These adverse events occur when immune system regulation is disrupted by ICIs, leading to symptoms similar to autoimmune diseases and affecting multiple organs, including the skin, respiratory system, nervous system, endocrine system, and digestive system. The incidence of ICI-induced diarrhea and colitis varies significantly between different drug classes. For PD-1/PD-L1 inhibitors, diarrhea and colitis occur in approximately 20% of patients, with severe toxicities (grade 3-4) affecting only 2%-5% of patients. In contrast, CTLA-4 inhibitors are associated with notably higher rates of gastrointestinal adverse events; ipilimumab induces gastrointestinal symptoms in approximately 40% of cases, with severe inflammation occurring in 10%-15% of patients [2, 3, 6]. This distinction is clinically relevant as it informs treatment selection and monitoring strategies. In this case, PD-1 inhibitors were used, making it a rare occurrence in terms of the frequency of severe disease. The symptoms of irAE colitis resemble those of ulcerative colitis; moreover, there is substantial overlap between irAE colitis and inflammatory bowel disease, both endoscopically and histologically. Therefore, the treatment of irAE colitis should be consistent with the treatment of ulcerative colitis.

Guidelines for irAEs have been published by the American Society of Clinical Oncology (ASCO), the European Society for Medical Oncology (ESMO), and other organizations. These guidelines recommend treatment strategies based on a certain level of consensus [2,3]. They suggest that patients with grade 2 or higher irAE colitis should discontinue ICI treatment and receive high-dose systemic corticosteroids. For steroid-resistant patients, infliximab is recommended. More recently, vedolizumab has been reported to be effective in cases that are refractory to steroids and infliximab. Mesalazine has also been reported to be effective for maintenance therapy [7]. Although surgical treatment for refractory irAE colitis is not specified in the guidelines, it has been managed in the same manner as ulcerative colitis. For irAE colitis with perforation, a subtotal colectomy with ileostomy and sigmoidoscopy is recommended because colonic lesions are generally extensive, and segmental colonic resection is often followed by severe inflammation of the remaining colon in the postoperative phase. However, some reports have documented deaths within a few days after subtotal colectomy [8]. Moreover, subtotal colectomy is considered highly invasive and high-risk for most patients with severe irAE colitis. In addition, case reports have described ileostomy following colonic perforation in irAE colitis, with a poor prognosis [9]. In contrast, some studies have reported that ileostomy is effective in managing refractory irAE colitis. Additionally, a case report described the use of a diverting loop ileostomy to treat Fournier's gangrene accompanied by refractory irAE colitis, with the patient experiencing an improvement in colitis [10, 11]. In this case, prednisolone, infliximab, and mesalazine did not significantly improve the symptoms, and leukocytapheresis was performed. Although medical treatment provided temporary symptom relief, irAE colitis recurred and became highly refractory to medical therapy. Therefore, surgical intervention was considered. However, the patient's performance status was 3. A subtotal colectomy was deemed too invasive and high-risk, so a less invasive surgical approach was chosen. Consequently, an ileostomy was performed to allow the colon to rest. Abdominal symptoms resolved in the early postoperative period, enabling the resumption of ICI treatment. However, a postoperative colonoscopy showed no improvement in the mucosal lesions, thus preventing stoma closure. The difference between irAE colitis and ulcerative colitis is that irAE colitis is a side effect of cancer treatment by ICIs. There is a conflicting view that discontinuing ICIs is preferable for severe irAE colitis; however, continuing ICIs is preferable from an oncological perspective. In other words, it is necessary to explore ways to avoid discontinuing ICIs in the treatment of irAE colitis, including surgical options. There is limited evidence on the safety of resuming treatment with ICIs in patients whose treatment was interrupted due to the onset of irAEs. In a report of 24,079 irAE cases registered in the World Health Organization VigiBase database, 452 patients were rechallenged with ICIs, and 130 (28.8%) of them experienced a recurrence of irAEs. Colitis, pneumonia, and hepatitis were reported to have high recurrence rates [12]. In another report of 167 patients with irAE colitis, one-third of those who were rechallenged with ICIs relapsed with irAE colitis [13]. It has been reported that the risk-reward ratio of anti-PD-1 or anti-PD-L1 rechallenge is within acceptable limits [14]. These findings suggest that rechallenging ICIs is feasible under close monitoring, although further studies are needed. Currently, the standard surgical procedure for treating ulcerative colitis is proctocolectomy. Appendicostomy, cecostomy, and ileostomy have been performed for ulcerative colitis since the early 1900s to restore colonic function. Ileostomy, in particular, was commonly used in patients with inflammatory bowel disease who developed toxic colitis. Subsequently, proctocolectomy became more widely adopted; however, it was associated with a high mortality rate due to intraoperative fecal spillage. In 1971, Turnbull et al. highlighted the risk of fecal contamination during colonic manipulation and proposed diverting ileostomy and decompressive colostomy as a solution [15]. Among these techniques, the Turnbull-Blowhole colostomy was designed to decompress the colon and has been reported to induce temporary disease remission in many patients. Although its use has declined with the advancement of medical therapies, it has recently attracted renewed attention and has been reported to be effective in managing toxic ulcerative colitis during pregnancy [16,17].

Colostomy has also been employed in non-ICI-related conditions, including inflammatory bowel disease, diverticulitis with perforation, and colorectal cancer. In severe cases of ulcerative colitis or Crohn’s disease, colostomy may serve as a bridge to definitive surgery or as a palliative measure. Historical use of decompressive colostomy for toxic megacolon and obstructive colitis further illustrates its utility in managing severe colonic inflammation, especially when more extensive surgery is not feasible. In the present case, colonic decompression was not required; therefore, a Turnbull-Blowhole colostomy was not performed. Nonetheless, this technique may represent a viable option for patients with irAE colitis complicated by toxic megacolon, particularly in those with poor general condition.

## Conclusions

With the increasing use of ICIs, clinicians must remain vigilant in recognizing, diagnosing, and effectively managing irAE colitis to ensure optimal patient outcomes. As the indications for ICIs continue to expand, the incidence of irAE colitis is expected to rise, making it crucial to explore various treatment strategies. This case underscores the importance of evaluating surgical interventions for refractory irAE colitis, particularly in patients who cannot tolerate more invasive procedures. Given the lack of standardized guidelines for surgical management, further research is essential to establish evidence-based recommendations and improve long-term patient care.
